# Charcoal Reflectance Reveals Early Holocene Boreal Deciduous Forests Burned at High Intensities

**DOI:** 10.1371/journal.pone.0120835

**Published:** 2015-04-08

**Authors:** Victoria A. Hudspith, Claire M. Belcher, Ryan Kelly, Feng Sheng Hu

**Affiliations:** 1 Department of Plant Biology, University of Illinois at Urbana-Champaign, Urbana, Illinois, United States of America; 2 PalaeoFire Lab, Department of Geography, Hatherly Laboratories, University of Exeter, Devon, United Kingdom; Chinese Academy of Sciences, CHINA

## Abstract

Wildfire size, frequency, and severity are increasing in the Alaskan boreal forest in response to climate warming. One of the potential impacts of this changing fire regime is the alteration of successional trajectories, from black spruce to mixed stands dominated by aspen, a vegetation composition not experienced since the early Holocene. Such changes in vegetation composition may consequently alter the intensity of fires, influencing fire feedbacks to the ecosystem. Paleorecords document past wildfire-vegetation dynamics and as such, are imperative for our understanding of how these ecosystems will respond to future climate warming. For the first time, we have used reflectance measurements of macroscopic charcoal particles (>180μm) from an Alaskan lake-sediment record to estimate ancient charring temperatures (termed pyrolysis intensity). We demonstrate that pyrolysis intensity increased markedly from an interval of birch tundra 11 ky ago (mean 1.52%Ro; 485°C), to the expansion of trees on the landscape ∼10.5 ky ago, remaining high to the present (mean 3.54%Ro; 640°C) irrespective of stand composition. Despite differing flammabilities and adaptations to fire, the highest pyrolysis intensities derive from two intervals with distinct vegetation compositions. 1) the expansion of mixed aspen and spruce woodland at 10 cal. kyr BP, and 2) the establishment of black spruce, and the modern boreal forest at 4 cal. kyr BP. Based on our analysis, we infer that predicted expansion of deciduous trees into the boreal forest in the future could lead to high intensity, but low severity fires, potentially moderating future climate-fire feedbacks.

## Introduction

The modern Alaskan boreal forest is sensitive to climate forcing [1], as shown by recent dramatic increases in the occurrence of late season, high severity fires [2–4]. Black spruce (*Picea mariana* (Mill.) B.S.P.) is the dominant species in the boreal forest [5]. Post-fire, black spruce typically follows a self-replacement trajectory [3] but sites that burn at high frequency or severity can alter this successional pattern by favoring the recruitment of highly competitive deciduous stands of aspen (*Populus*), birch (*Betula*) and willow (*Salix*) [6–7]. As such, it is generally accepted that the vegetation composition of boreal forests will change in response to future anticipated anthropogenic warming [2, 3, 6, 8, 9]. In particular, climate-driven changes in fire regime will likely result in an expansion of deciduous species, which will have additional impacts on permafrost dynamics and soil organic carbon storage [10].

Millennial-scale shifts in vegetation composition in Alaska have been shown to alter the flammability of the landscape throughout the Holocene [11–12]. The fire regime in the modern boreal forest is characterized by large, high intensity wildfires [13], and has been shaped by the expansion of fire-adapted black spruce on the landscape 4–6 ky ago [1]. Yet, deciduous forest cover dominated this region between 10–9 ka [14]. Deciduous vegetation generally has a higher fuel moisture content than spruce [2] and is therefore likely to burn less intensely than spruce [15]. Therefore, the predicted future expansion of deciduous forest may moderate the intensity with which fires will burn in this ecosystem. Vegetation-driven variations in fire intensity are also likely to have a strong feedback on fire severity, resulting in significant implications for carbon cycling in the boreal forest.

**Fig 1:**
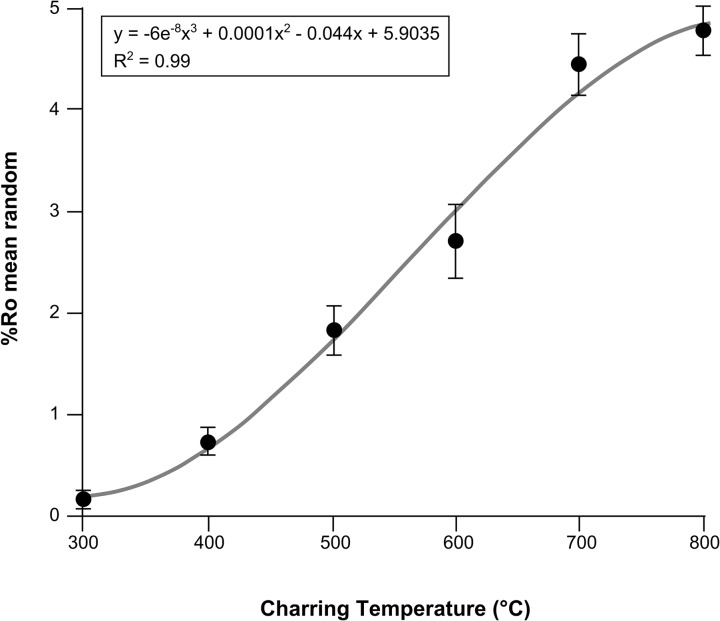
Combined charcoal reflectance calibration curve for five experimentally charred boreal woods (*Betula nana*, *Picea mariana*, *Picea glauca*, *Betula papyrifera*, *Populus tremuloides*). Mean random reflectance under oil (Ro_mean_) and standard deviations represent all species.

Paleorecords have been used to infer fire regime shifts in response to changing vegetation in Alaska throughout the Holocene [11, 16–17] but are currently unable to link vegetation changes to aspects of fire behavior, such as intensity, due to a lack of fire intensity proxy. Charcoals can be analyzed using reflected light microscopy, where measurements of the amount of light reflected from a charred sample have been shown to correspond to the temperature of formation of the charcoal [18] (Fig. 1). Charcoal is a product of the thermal decomposition of organic matter in the absence of oxygen (pyrolysis) during combustion events. Therefore, we consider charcoal reflectance to represent the minimum temperature plant material is heated to during the pyrolysis stage of combustion, and is herein termed pyrolysis intensity. Here we apply this technique to Holocene charcoals to elucidate whether vegetation shifts throughout the Holocene have influenced fire intensity, by using pyrolysis intensity as a proxy for past fire intensity. The application of this technique to Holocene charcoals is novel, and yields new information about past fire regimes previously unavailable by using the quantification of charcoal abundance alone.

Fire intensity is a key component of fire regimes, and charring temperature in particular, may relate to vegetation mortality post-fire. Mortality is also an aspect of fire severity, which records the above- and below-ground loss of organic matter following a fire [19]. Therefore, future climate-driven vegetation change in the boreal forest is likely to influence both fire intensity and severity in this ecosystem. We aim to assess the extent to which spruce alone can be linked to high fire intensity and whether past intervals of mixed spruce-deciduous vegetation are also capable of burning at high intensities. Modern black spruce relies on high intensity crown fires to aid seed dispersal from semi-serotinous cones, whereas post-fire recruitment of aspen is typically higher in areas of low fire intensity [20], therefore variations in fire intensity should have direct ecological feedbacks.

**Fig 2:**
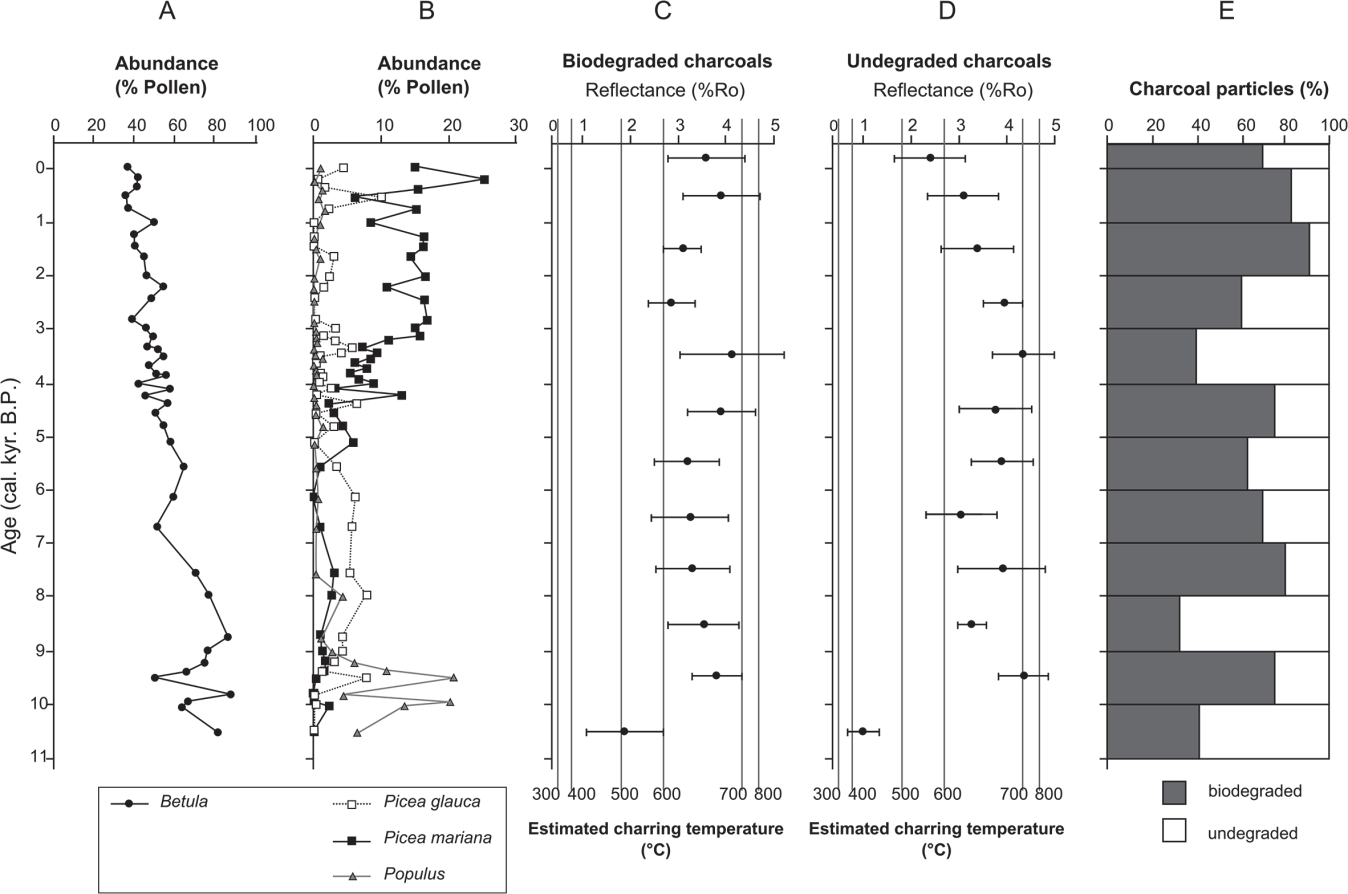
Vegetation and pyrolysis intensities from Screaming Lynx Lake, during the Holocene, (A) birch pollen (primarily *B*.*nana*) percentages, (B) pollen percentages of main tree species referred to in the text (modified from ref. 17). Random-effects reflectance estimates for, (C) biodegraded charcoals, and (D) undegraded charcoals (both plotted with 95% confidence intervals), (E) Proportions of biodegraded and undegraded charcoals from each 1kyr interval.

To assess variations in fire intensity throughout the Holocene we measured pyrolysis intensities of charcoal from Screaming Lynx Lake. This lake is within the Yukon Flats, one of the most flammable ecoregions in the modern Alaskan boreal forest [17]. The sedimentary record in this study encompasses the last 10.6 cal. kyr BP [17], spanning several major vegetation zones, as inferred from pollen analysis [16]: 1) birch (*Betula*) tundra (10–11 cal. kyr BP); 2) mixed deciduous (*Populus*) and white spruce (*Picea glauca*) woodland (9–10 cal. kyr BP); and 3) expansion of black spruce (*Picea mariana*) resulting in the establishment of the modern boreal forest (∼4.5 cal. kyr BP) (Fig. 2A-B). Our new approach to quantifying pyrolysis intensity across these millennial-scale biome shifts offers an opportunity to assess how the changes in vegetation dominance link to variations in fire intensity.

## Materials and Methods

### Ethics statement

No permits were required for the described study, which complied with all relevant regulations. The fresh branch samples of *Populus tremuloides* (trembling aspen), *Betula papyrifera* (paper birch), *Picea mariana* (black spruce), and *Picea glauca* (white spruce) were sampled from the Morton Arboretum, Lisle, Illinois, USA (41.8169°N, 88.0688°W) by their arborist, David Marin. The *Betula nana* (dwarf birch) samples were obtained from the Royal Botanic Gardens, Edinburgh, UK and sampled by their horticulturalist Peter Brownless. None of the material used is listed as endangered or threatened by the IUCN.

### Study site and sample preparation

Screaming Lynx Lake (66° 04′N, 145° 24′W, 223m above sea level), interior Alaska, was originally sampled in 2007 [21]. Two overlapping cores were taken using a modified Livingstone corer and a polycarbonate piston corer was used to sample the sediment-water interface. The cores were correlated using lithological markers and magnetic susceptibility. Sediments were subsampled at 0.25cm resolution. Site description, pollen and charcoal sampling techniques, the age-depth model and fire frequency methods are all given in refs. 17 and 21.

To assess variations in fire intensity throughout the Holocene we obtained pyrolysis intensities from macroscopic charcoal particles (>180μm). Samples (n = 32 in total) were selected from charcoal peaks in the sedimentary record that were previously interpreted to represent local fire events [17]. Samples were evenly spaced throughout the record to encompass the vegetation zones throughout the Holocene (see Fig. 2A,B). All charcoal particles in a given sample were picked by hand and mounted on a glass cover slip, embedded in epoxy resin and polished.

### Charcoal reflectance

Charcoal is a relatively recalcitrant, slowly cycling organic compound [22] that can be preserved in soils, sediments and rocks for millennia. In addition, the molecular structure of charcoal becomes more aromatic with increasing temperature of formation [23]. When studied under oil, using reflectance microscopy, this increase in ordering of the charcoal structure with increasing charring temperature, translates to a predictable increase in the measurable amount of light reflected from the sample [24]. This well-established relationship derives from experimentally producing charcoals at known temperatures and for known durations in order to generate calibration curves (i.e. Fig. 1). Despite the artificial production of charcoal under these controlled experimental conditions; there is reasonable agreement between the interpreted charring temperatures of wildfire-derived charcoal extrapolated from these calibration curves and actual measured temperatures from modern boreal wildfires e.g. [25–26]. Reflectance measurements of wildfire-derived charcoal can therefore provide an indirect measure of the minimum temperature plant material has been subjected to during a wildfire e.g. [18, 24, 26–28]. We experimentally produced charcoals of representative boreal (tundra and forest) vegetation observed throughout the Screaming Lynx sedimentary record (Fig. 2A-B), for a variety of temperatures. Fresh wood samples of *Populus tremuloides* (trembling aspen), *Betula nana* (dwarf birch), *Betula papyrifera* (paper birch), *Picea mariana* (black spruce), and *Picea glauca* (white spruce) were cut into 15mm sized pieces (bark intact), wrapped in foil and heated in steel containers e.g. [18, 28] in a Carbolite furnace at the University of Exeter. The wood samples were heated at 100°C temperature increments from 300°C—800°C, each for 1h duration to replicate the likely temperature range experienced in wildfires. The resulting charcoals were embedded in epoxy resin and polished.

Reflectance measurements for modern experimental and Holocene charcoals were obtained using a Leica DM2500P reflectance microscope at Southern Illinois University Carbondale, USA. The reflectance microscope was calibrated using a series of synthetic standards, ST (5.460%Ro), CZ (3.170%Ro), GGG (1.717%Ro) and Glass (0.940%Ro), using TIDAS MSP software. Images were taken using a Leica DFC 400 digital camera. Three replicates were analyzed per temperature for the experimental wood charcoals, with one hundred random measurements taken across the late wood cells in each sample. This produced the calibration curves shown in Fig. 1. Mean charcoal reflectance is strongly correlated with production temperature, as shown in Fig. 1 for experimentally charred woods from the five boreal tree species (R^2^ = 0.99) following the polynomial function:

$$y=\;-\mathrm{6.0}\;\times \;{\mathrm{10}}^{-8}x^{3}\;+\;\mathrm{1.0}\;\times \;{\mathrm{10}}^{-4}x^{2}\;-\;\mathrm{4.4}\;\times \;{\mathrm{10}}^{-2}x\;+\;\mathrm{5.9}$$ (1)

Where *y* is the formation temperature (°C) and *x* is the charcoal reflectance value (%Ro).

For the Holocene charcoals, 30 random unbiased reflectance measurements made per particle, with a minimum of 5 particles measured per sample. We converted these reflectance values to estimates of pyrolysis intensity using the combined calibration curve in Fig. 1. As this is the first application of the reflectance technique to charcoal from a Holocene lake record, the production of polished blocks containing charcoal particles >180μm in size is still in its infancy, and sample loss during polishing prevented more charcoal particles being measured. Nonetheless, there is currently no definitive methodology for determining the minimum number of particles required to statistically represent a wildfire-derived charcoal assemblage and previous published work has reported a wide range of measurements from, 50 [29] to 100 [18] per sample. Moreover, a recent study by ref. 30 has demonstrated that comparable fuel types char at similar temperatures during a wildfire. In this study, we observed similar fuel types throughout this lake record (primarily wood and needles) and as such, we believe that ≥150 measurements per sample encompasses the likely range of potential reflectance values for all the charcoal in a given sample.

**Fig 3:**
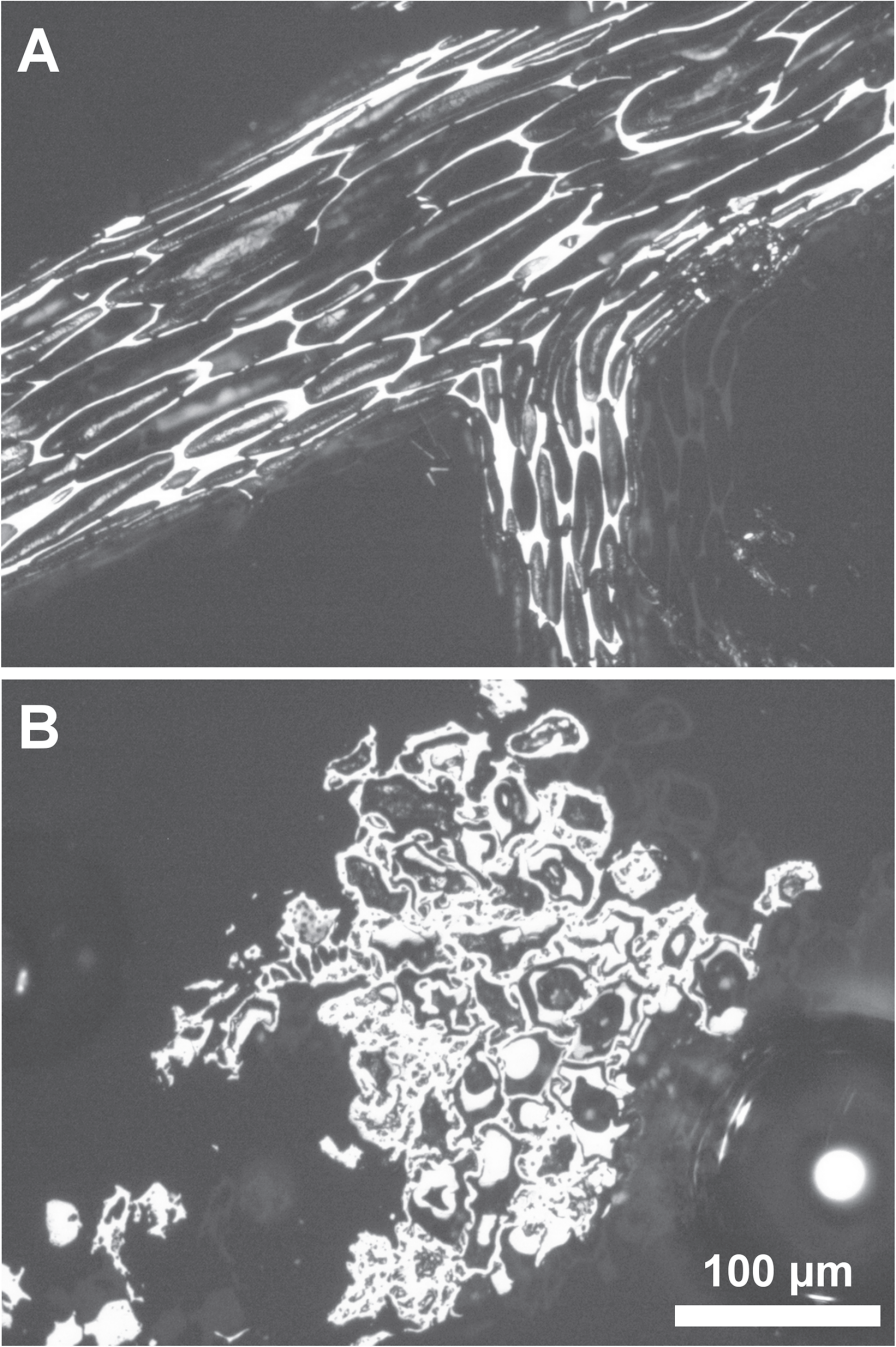
Photomicrographs illustrating charcoals from ‘undegraded’ and ‘biodegraded’ categories, (A) undegraded gymnospermous woody fragment, (B) Plant tissue showing evidence of biodegradation prior to charring: the presence of cell infillings formed from biodegradation of the cell wall, distortion of cell walls, and cavity formation in secondary walls. Photographs were taken using reflected-light microscopy with immersion oil.

In addition to measured reflectance, each Holocene charcoal particle was classified as ‘biodegraded’ or ‘undegraded’ based on whether the charcoal showed characters indicative of decay prior to charring [31] (Fig. 3B). Plant material in the litter layer commonly shows signs of biodegradation [32], therefore biodegraded charcoals (e.g. Fig. 3B) likely derive from surface fuels, hence represent litter fires; whereas undegraded charcoals (e.g. Fig. 3A) likely derive from crown fuels hence indicate crown fires. It is of note that undegraded charcoals primarily comprised unaltered woody plant material (illustrated in Fig. 3A) and spruce needles; however, roots and mosses can also be undegraded when charred, yet represent surface fuels. Neither were observed in this study.

We consider pyrolysis intensity to represent the charring temperatures reached during the pyrolysis stage of combustion in flaming fires [30]. High intensity crown fires generally consume the majority of needles, or convert them to ash [33]. The preservation of charred needles in this lake-sediment record therefore suggests that we are capturing the highest pyrolysis intensities experienced during these wildfires.

### Random-effects model

Statistical analysis was conducted by RK, using R software (version 3.1.1) [34]. The reflectance data were analyzed using a random-effects model using the ‘lme4’ package in R, whereby differences among samples were considered fixed effects, and differences among particles within a sample were represented as random effects. Resulting estimates of mean reflectance for each sample are plotted with 95% confidence intervals (Fig. 2C-D).

### Calorimetry experiments

**Fig 4:**
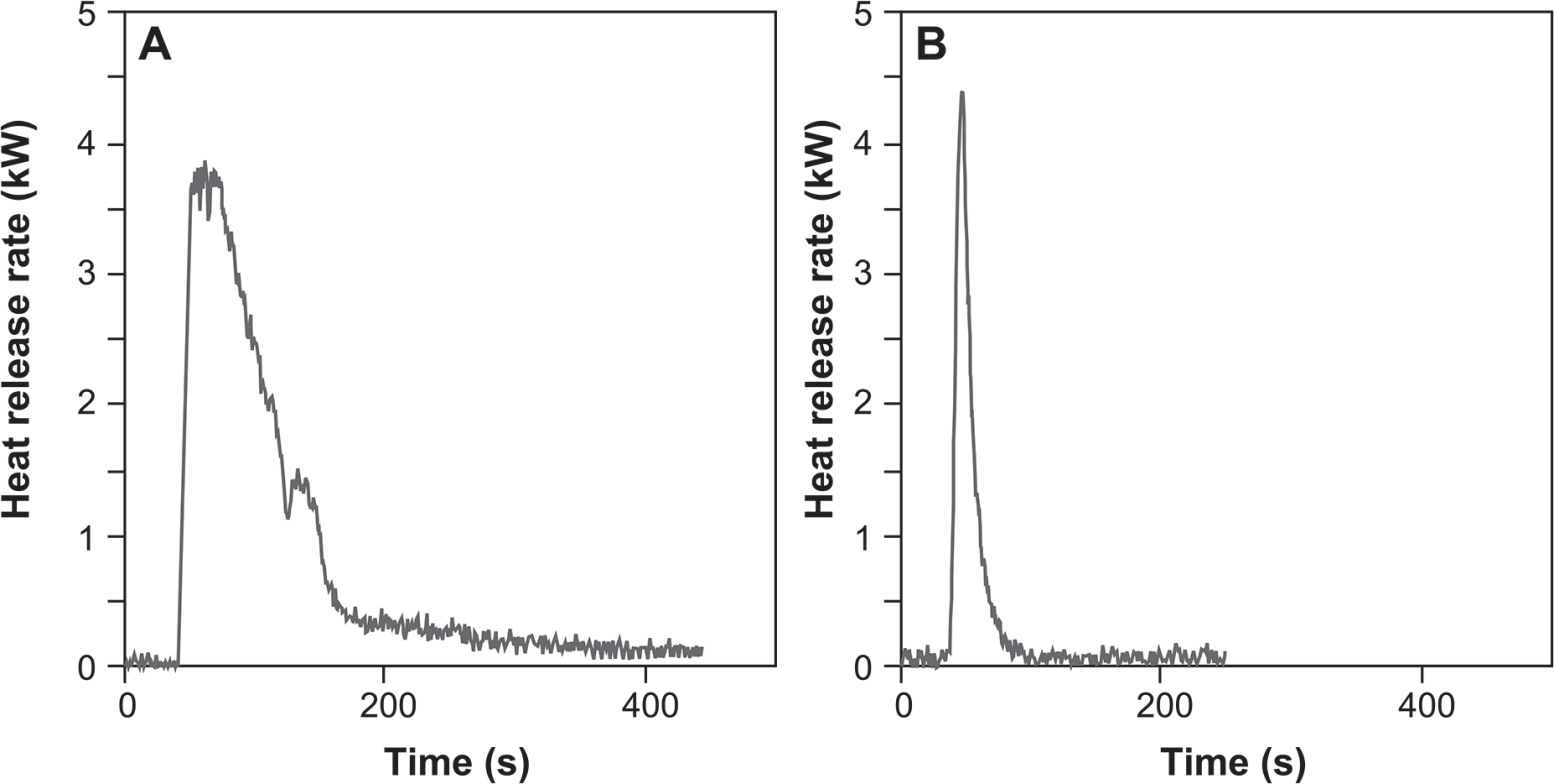
Total heat release curves for (A) Spruce (*Picea* sp.) needle litter and (B) Aspen (*Populus* sp.) broadleaf litter.

Aspen (*Populus* sp.) and spruce (*Picea* sp.) leaf litter were burned using a cone calorimeter to ascertain the burn duration and total heat release of both needle and broadleaf litter (Fig. 4). The cone calorimeter apparatus represents an international standard (ISO 5660-Part 1; ASTM E1354) in experimental fire testing of the heat release rates of materials [35]. The amount of oxygen consumed during combustion has been shown to be proportional to the heat released from the fuel [36]. Therefore, the cone calorimeter measures the exhaust gases from the combustion of the fuel to calculate the heat release rate [37]. The fuel samples were exposed to a radiant heat flux of 30 kWm^-2^, in order to replicate the likely lower limit of radiant heat fluxes from active surface fires in boreal forests e.g. [38]. Pyrolysis gases are driven off the heated fuel sample and react with air to generate a flammable mixture. This is ignited by a spark igniter, which in turn ignites the solid fuel [39].

The leaf litter samples were first dried at 50°C for six days, until 5% moisture had been attained in order to remove the effect of fuel moisture on foliar flammability. A 3cm depth fuel bed was created for each species by filling a 368cm^3^ metal mesh basket according to the natural packing density of the leaves [40] to ensure equal litter volume was analyzed for each species. Three replicates were analyzed per species.

## Results and Discussion

The reflectance values presented here represent 6,390 measurements (213 charcoal particles were measured from 32 samples. Of these, 4,590 measurements were on biodegraded, and 1,800 on undegraded charcoals; illustrated in Fig. 3). The fire regime in the modern boreal forest comprises both surface and crown fires [41]. Surface fires consume understory vegetation (preserved as undegraded charcoals), as well as decaying plant material in the litter and duff (biodegraded charcoals; Fig. 3B), whereas crown fires consume finer fuels e.g. twigs, needles, leaves (undegraded charcoals; Fig. 3A). In vegetation zones dominated by low-lying fuel, such as tundra, combustion of live, small-stature birch shrubs will also produce undegraded charcoals. Surface litter is not required for fire propagation and these fires may also be considered as crown fires [42]. The majority of charcoal particles preserved in the Holocene sediments of Screaming Lynx Lake represent biodegraded charcoals (72% of all measurements). However, biodegraded and undegraded charcoals were observed in all samples, at each time interval, providing evidence for combustion of surface and crown fuels throughout the Holocene (Fig. 2C-D).

In this record, we observe a singular period of low pyrolysis intensity (1.17%Ro undegraded, 1.87%Ro biodegraded; 450°C and 510°C respectively; Fig. 2C-D), during an interval of birch shrub tundra (10–11 cal. kyr BP; Fig. 2A). Modern tundra fires are typically of low- to moderate- intensity [5] and low charcoal reflectance values (corresponding to charring temperatures <500°C) have also been recorded in modern tundra fires of northwestern Alaska (VH, CMB, FSH personal observations).

Following the birch tundra interval, pyrolysis intensity increased dramatically from mean 1.52%Ro (485°C) to mean 3.54%Ro (640°C), for both biodegraded and undegraded charcoals, coincident with the establishment of trees on the landscape < 10 cal. kyr BP, as inferred from a marked increase in arboreal pollen at 10.5 cal. kyr BP (Fig. 2B). The initial vegetation composition consisted of mixed stands dominated by aspen, with scattered white spruce, at 9–10 cal. kyr BP (Fig. 2B) and exhibited some of the highest pyrolysis intensities in the entire record for both surface and crown fuels (4.35%Ro undegraded, 3.81%Ro biodegraded; corresponding to 665°C -720°C; Fig. 2B-D). Aspen has low flammability due to high inherent moisture content of the fine fuels [2], and reduced surface fuel loads due to relatively rapid decomposition of aspen compared to spruce litter [32]. Yet, the majority of charcoals from this interval derive from surface fuels (Fig. 2E). Despite rapid litter decomposition rates, broadleaved species (such as aspen) produce a low bulk density surface litter [32], which enhances heat diffusion during fires and enables rapid spread of surface fires; the flammability of which would have been further enhanced by the addition of spruce needles [43]. Such litter flammability characteristics may also provide an indication of potential past burn severity. By measuring the heat release rate and burn duration of spruce and aspen needle/leaf litter (Fig. 4) we have demonstrated that both spruce and aspen attain comparable peak heat release rates (Fig. 4), validating the high pyrolysis intensities observed for both species (Fig. 2C,D). Yet, the aspen litter shows shorter burn duration than spruce (Fig. 4); short duration, high intensity burns typically limit heat transference to the soil [44] suggesting that surface fires during this interval may have been of high intensity, but low severity, hence less ecologically damaging than surface fires in spruce-dominated boreal forest.

In addition, it is known that mixed stands of conifer and aspen are capable of carrying high intensity crown fires, which can lead to stand-replacing burns [45]. Samples from the 9–10 cal. kyr BP interval contained charred spruce needles, despite correspondingly low spruce pollen percentages (Fig. 2B), suggesting high intensity crown fires were also part of the fire regime during this time interval.

The decrease in the abundance of aspen pollen around 9 cal. kyr BP (Fig. 2B) coincided with an increase in white spruce, and high but decreasing abundance of birch pollen percentages (Fig. 2A-B). These changes suggest an ecological transition to an open forest-tundra ecosystem with diminished abundance of aspen. Pyrolysis intensities continue to remain high in the 8–9 cal. kyr BP interval (Fig. 2C-D), with a shift from surface to crown fire regime (Fig. 2E). The presence of charred spruce needles during this interval suggests that the high fire intensities were driven by the expansion of white spruce on the landscape.

The pyrolysis intensities of surface and crown fuels remained high in all subsequent intervals to the present, irrespective of vegetation composition (Fig. 2). The expansion of black spruce associated with the establishment of the modern boreal forest (∼4.5 cal. kyr BP) markedly increased both fire frequency and biomass burned in Alaska [17]. In this study, the expansion of black spruce at 4 cal. kyr BP is represented by high pyrolysis intensities (4.35%Ro undegraded, 4.12%Ro biodegraded; corresponding to 695°C -720°C; Fig. 2B-D) that are comparable to the 9–10 cal. kyr BP interval of mixed aspen-spruce vegetation. Yet, unlike the 9–10 cal. kyr BP interval, we have shown that spruce litter burns intensely (high heat flux) for long durations (Fig. 4A), suggesting these high intensity surface fires at the 4 cal. kyr BP interval would also result in higher burn severity. Black spruce forests provide continuous fuel distribution, from understory shrubs to low-lying branches all of which greatly enhance crown fire potential [1], as evidenced in this record by a shift from combustion of surface to predominantly crown fuels (Fig. 2E). Reflectance estimates differ between biodegraded and undegraded charcoal particles at 0–1 and 2–3 cal. kyr BP (Fig. 2C-D). At 2–3 cal. kyr BP reflectance values are higher for undegraded than for biodegraded charcoals suggesting higher intensity crown fires. In contrast, biodegraded charcoals from 0–1 cal. kyr BP are more abundant (Fig. 2E) and yield higher reflectance values, suggesting a shift to high pyrolysis intensity surface fires (Fig. 2C,D). This may be related to an increase in deciduous species in the study area, as evidenced by early successional deciduous forests and mixed-forest classes on the landscape [17], suggesting that recent climate-driven changes in fire regime appear to be altering post-fire successional trajectories.

Our results indicate that abundant deciduous forest cover from 9–10 cal. kyr BP led to short duration, high intensity fires. Such a transition today would therefore regulate fire severity in the boreal forest and the current short term positive climate forcing caused by massive carbon release during high severity fires in spruce forests [4] may be offset in the long term by the shift in vegetation dynamics favoring high intensity, but low severity fires in deciduous dominated forests.

## Conclusion

Our novel application of using reflectance microscopy of charcoals to interpret pyrolysis intensity, coupled with modern experiments, provides an additional facet in interpreting modern and ancient fires. Pyrolysis intensities in this record have remained high since the transition from tundra to forest and the expansion of trees on the landscape at ∼10.5 ky ago. Our study reveals that the highest pyrolysis intensities in this record derive from periods of vegetation transition: mixed aspen-white spruce woodland at 10 cal. kyr BP and the expansion of black spruce at 4–5 cal. kyr BP (Fig. 2C-D). High pyrolysis intensities observed for mixed stands in the early Holocene suggests that if climate-driven succession favors deciduous encroachment into boreal forest (forming mixed-stands), as is suggested by current models for the future [2–3], then fires in this ecosystem will likely continue to be of high intensity, but lower severity. Our findings suggest that future research needs to consider the likely impact of high intensity fires, that burn for shorter durations, in encroaching mixed forest stands and how this may influence long-term carbon cycling in the boreal forest.
